# Supplementary material for: Whole brain and deep gray matter structure segmentation: Quantitative comparison between MPRAGE and MP2RAGE sequences
Source: PLoS One. 2021 Aug 6;16(8):e0254597. doi: 10.1371/journal.pone.0254597 (PMC8345829; doi:10.1371/journal.pone.0254597)
Supplement: Attachment — Submitted filename: Reviewers_comments.docx [file pone.0254597.s002.docx]

**Reply to Reviewer's comments**

Reviewer #2: I appreciate the authors' efforts to accommodate most of my concerns, which has improved the manuscript. I like the new Figure 3, which nicely shows the differences for the GM, WM and CSF segmentations between acquisitions for each method. WM and CSF are generally larger based on MPRAGE data using FSL-FAST (especially) and volBrain, while more similar using CAT12. The opposite is true for GM. In addition, I thank the authors for calculating CNR per lobe and I think this is informative and should be added to the main text.

**We thank the reviewer for his valuable efforts and constructive feedback and suggestions. We added the CNR per lobe to the text.** *Furthermore, we added the range of variation of CNR per lobe as suggested to the results section.*

Finally, I recommend further clarification of a couple of remaining issues:
1. The authors state that 'MP2RAGE resulted in more accurate brain tissue segmentation' (abstract). This needs to be rephrased as the 'ground truth' is not known so it is difficult to define 'more accurate', or, it should be clarified what they mean with 'more accurate'.

**Response:** We thank the reviewers for this comment. Indeed, the use of the phrase "more accurate" here is not accurate. Therefore, we replaced it with "reproducible", as supported by the results of test- retest study. We also changed this in the conclusions paragraph in the discussion.

2. I think the difference between the MPRAGE and MP2RAGE sequences need to be emphasized more. Especially in terms of how the T1w-like image is obtained (i.e., synthesized) for the MP2RAGE sequence.

**Response:** We have modified the third paragraph describing MP2RAGE in the introduction, further clarifying how the MP2RAGE image is acquired and synthesized.

3. In their response to my comment #3, the authors provided an intra-subject difference map based on the segmentation results. Although informative as well, what I was suggesting was to calculate the difference between the raw images. This allows characterization of any spatial bias in the difference between MPRAGE and MP2RAGE data. For example, are differences larger towards the frontal lobe?

**Response:** We apologize for the misunderstanding of this point in the previous round. Since MPRAGE is not a quantitative sequence, and due to the difference in dynamic range between both sequences comparison of the raw images might not be meaningful (or accurate). Nevertheless, to perform the spatial comparison as suggested we first normalized both images relative to the white matter signal, and then we calculated the differences between the normalized images (See Figure Rev1. below). As it can be seen in the figure, following normalization only minor differences were demonstrated between the sequences for the WM and GM tissues (<±0.25, normalized values).

**Figure Rev1:**

4. Please verify that CNR values were calculated using the raw T1w images, and elaborate on what the potential impact of readout bandwidth differences between MPRAGE and MP2RAGE acquisitions on CNR could be.

**Response:** We confirm that CNR was calculated on the acquired raw T1 images. We clarified this point in the corresponding section in the methods. MP2RAGE has higher bandwidth compared to MPRAGE (240 Hz/Px vs. 150 Hz/Px). The use of high bandwidth is expected to reduce susceptibility effects including eddy currents associated with metal, and thus to improve image SNR [See: Marquess et al., 2010, Neuroimage]. We added this information to the discussion.

5. I would advise to add the T-statistical value and degrees of freedom to the statistical results. Also, specify that the non-binary probability masks were used and if statistical testing was restricted to GM and/or WM (i.e., masked), or not.

**Response:** We now added the T statistical values in the corresponding sections in the results as suggested. Also, in ***Statistical analysis*** sub-section of the Methods, we section further clarified that the statistical testing was performed on the masked GM and WM probability maps achieved by the different used segmentation tools.

Figures:
6. Figure 1, if possible, I would recommend to use data from the same subject for all sub-panels.

**Response:** All the images in the sub-panels in Figure are actually from the same subject. As noted by the reviewer, slight variations in the slices were found however, we now rectified this and made sure that all slices match.

7. Figure 4, it is very difficult to see the significant voxels. I would advise to zoom in on the interesting part and only show that in the figure.

**Response:** We agree with this comment. We have reduced the number of slices shown in the left panel to better depict the significant voxels in GM and WM.

Some minor textual comments I noticed:
8. Abstract: 'In a sub-study, twelve participants were scanned after one year.' change to ' rescanned'

**Response:** This was changed as suggested.

9. Abstract: 'Mean ± SD = 0.97 ± 0.04 and 0.8 ± 0.1 respectively; p<0.0001' change 'and' to 'vs'

**Response:** The word and was changes to VS.

10. Introduction, last paragraph: 'different widely available, open-source automatic segmentation tools' remove open-source. I was not able to find the source code for volBrain

**Response:** We omitted the phrase "open-source" as advised.

11. Methods, CNRwg: 'corrected per scan time' change to 'normalized for scan time'

**Response:** This was changed accordingly.

12. Please go through the manuscript to correct small grammatical errors.

**Response:** We went over the manuscript thoroughly once again and corrected some minor mistakes. Hopefully we got them all!
